# Excessive Erythrocytosis and Cardiovascular Risk in Andean Highlanders

**DOI:** 10.1089/ham.2017.0123

**Published:** 2018-09-18

**Authors:** Noemí Corante, Cecilia Anza-Ramírez, Rómulo Figueroa-Mujíca, José Luis Macarlupú, Gustavo Vizcardo-Galindo, Grzegorz Bilo, Gianfranco Parati, Jorge L. Gamboa, Fabiola León-Velarde, Francisco C. Villafuerte

**Affiliations:** ^1^ Laboratorio de Fisiología Comparada, Departamento de Ciencias Biológicas y Fisiológicas, Facultad de Ciencias y Filosofía, Universidad Peruana Cayetano Heredia. Lima, Perú.; ^2^ Department of Medicine and Surgery, University of Milano-Bicocca, Milano, Italy.; ^3^ Department of Cardiovascular, Neural and Metabolic Sciences, IRCCS Istituto Auxologico Italiano, Milano, Italy.; ^4^ Division of Clinical Pharmacology, Vanderbilt University Medical Center, Nashville, Tennessee.

**Keywords:** andean highlanders, cardiovascular risk, chronic mountain sickness, excessive erythrocytosis

## Abstract

Corante, Noemí, Cecilia Anza-Ramírez, Rómulo Figueroa-Mujíca, José Luis Macarlupú, Gustavo Vizcardo-Galindo, Grzegorz Bilo, Gianfranco Parati, Jorge L. Gamboa, Fabiola León-Velarde, and Francisco C. Villafuerte. Excessive erythrocytosis and cardiovascular risk in Andean highlanders. *High Alt Med Biol.* 19:221–231, 2018.—Cardiovascular diseases are the main cause of death worldwide. Life under high-altitude (HA) hypoxic conditions is believed to provide highlanders with a natural protection against cardiovascular and metabolic diseases compared with sea-level inhabitants. However, some HA dwellers become intolerant to chronic hypoxia and develop a progressive incapacitating syndrome known as chronic mountain sickness (CMS), characterized by excessive erythrocytosis (EE; Hb ≥21 g/dL in men, Hb ≥19 g/dL in women). Evidence from HA studies suggests that, in addition to CMS typical signs and symptoms, these highlanders may also suffer from metabolic and cardiovascular disorders. Thus, we hypothesize that this syndrome is also associated to the loss of the cardiometabolic protection observed in healthy highlanders (HH), and therefore to a higher cardiovascular risk (CVR). The aim of the present work was to evaluate the association between EE and CVR calculated using the Framingham General CVR Score and between EE and CVR factors in male highlanders. This cross-sectional study included 342 males from Cerro de Pasco, Peru at 4340 m (HH = 209, CMS = 133). Associations were assessed by multiple logistic regressions adjusted for potential confounders (BMI, pulse oxygen saturation and age). The adjusted models show that the odds of high CVR (>20%) in highlanders with EE was 3.63 times the odds in HH (CI 95%:1.22–10.78; *p* = 0.020), and that EE is associated to hypertension, elevated fasting serum glucose, insulin resistance, and elevated fasting serum triglycerides. Our results suggest that individuals who suffer from EE are at increased risk of developing cardiovascular events compared with their healthy counterparts.

## Introduction

Cardiovascular diseases are the main cause of death worldwide (WHO, 2016), leading to the need for population-specific evaluation of cardiovascular risk (CVR) factors around the globe. Studies at high altitude (HA) have suggested that life in chronic hypoxia confers highlanders with a natural protection against the development of cardiovascular conditions such as hypertension (Hurtado, 1960; Marticorena et al., 1969; Negi et al., 2012), myocardial infarction, and coronary ischemic disease (Mortimer et al., 1977; Faeh et al., 2009); as well as against CVR factors such as diabetes (Picon Reategui, 1963; Woolcott et al., 2015) and dyslipidemia (Bellido et al., 1993; Baracco et al., 2006). However, a significant part of the HA population show reduced capacity to tolerate life in chronic hypoxia and develop excessive erythrocytosis (EE) and chronic mountain sickness (CMS). For this reason, CMS is considered to reflect nonadaptation to the HA environment.

CMS is a progressive incapacitating syndrome characterized by EE (Hb concentration ≥21 g/dL in men and ≥19 g/dL in women), severe hypoxemia, and by signs and symptoms such as headache, dizziness, breathlessness and/or palpitations, sleep disturbances, physical and mental fatigue, distended veins, and localized cyanosis (León-Velarde et al., 2005). Globally, 5%–10% of the ∼140 million people living above 2500 m develop EE and CMS (León-Velarde et al., 2005). In Peru, where about a third of the population lives at HA (INEI, 2007), epidemiological studies in the Andes above 4000 m report CMS prevalence between 15% and 20% in the adult male population. CMS prevalence increases with age, reaching 30% by the sixth decade of life (Monge-C et al., 1992). This syndrome not only represents a major health problem for highlanders but also hinders their occupational life.

Evidence suggests that in addition to the CMS symptomatology, these individuals may also suffer from metabolic and cardiovascular disorders that could contribute to a greater CVR and worsen their health status and living conditions (León-Velarde and Arregui, 1994; Jefferson et al., 2002; Okumiya et al., 2010, 2011, 2016; Gonzales and Tapia, 2013; De Ferrari et al., 2014). Studies in HA populations of Asia and the Andes have shown an association between elevated Hb concentration (>18 g/dL) and glucose intolerance (Okumiya et al., 2010, 2011, 2016), indicating a poor glycemic control in individuals presenting abnormally high Hb values for the altitude of residence (Okumiya et al., 2010, 2011, 2016). Also, a positive correlation was observed between increasing Hb concentration and conventional systolic blood pressure (SBP) and diastolic blood pressure (DBP) values in Cerro de Pasco, Perú at 4340 m (Gonzales and Tapia, 2013). Moreover, in studies at the same location, highlanders with EE showed a higher prevalence of hypertension, compared with their healthy counterparts (León-Velarde and Arregui, 1994; Jefferson et al., 2002). Although lipid metabolism has not been thoroughly investigated in individuals with EE, recent studies report an association between high Hb concentration and triglyceride levels (Gonzales and Tapia, 2013), and a higher prevalence of metabolic syndrome in the CMS population compared with healthy HA natives (De Ferrari et al., 2014).

Elevated hematocrit (Hct) also contributes to increased blood viscosity and thus, to higher risk of occlusive vascular disease and coronary heart disease (Danesh et al., 2000). Furthermore, elevated Hct might play a role in the development of metabolic disorders in individuals with EE through its effect on blood viscosity (Hanley et al., 2009). These findings suggest a negative effect of elevated red blood cell count on cardiometabolic parameters and CVR. However, to date, no study has specifically evaluated the risk of cardiovascular events in individuals with EE. We hypothesize that EE is associated to an increased CVR compared with healthy HA natives.

Underdiagnosis of high CVR and of the associated cardiometabolic alterations is common in developing countries (Gakidou et al., 2011) and is related to a lack of appropriate public health policies (WHO, 2014). Given that cardiovascular diseases are a major cause for mortality, especially in developing countries such as Peru (WHO, 2014, 2016), and CMS is a highly prevalent syndrome in Latin American highlanders, it is crucial that the cardiometabolic status of CMS patients is fully understood so that accurate and efficient strategies for the prevention and treatment of cardiometabolic disorders and increased CVR can be developed for this population. The aim of the present study was to assess the association between EE and high CVR, as well as between EE and individual CVR factors. In addition, we evaluated the association between EE and 24 h blood pressure measured by Ambulatory Blood Pressure Monitoring (ABPM).

## Methods

### Study design and population

This analytic cross-sectional study enrolled 209 healthy highlanders (HH) and 133 individuals with EE, all native and permanent residents of Cerro de Pasco, Peru, at 4340 m. Permanent residence at HA was defined as life-long permanence in the city with total time spent at a lower altitude shorter than 1 year. All participants were males between 18 and 66 years old. A nonprobabilistic convenience sampling method was used for recruitment of all participants. To this end, a Hct and blood pressure control campaign for the general Cerro de Pasco population was offered and advertised through radio broadcasting and through print advertising distributed throughout the city. Exclusion criteria were previous history of cardiovascular, respiratory or renal diseases, blood transfusions or phlebotomies in the previous 6 months, journeys to lower altitude for more than 7 days during the previous 6 months, and abnormal cardiac or pulmonary function evaluated through electrocardiogram and spirometry, respectively. Subjects with Hct ≥63% (Hb concentration ≥21 g/dL) were selected as participants with EE (León-Velarde et al., 2005), while subjects with Hct ≤58.5% were selected as healthy HA natives. The study was approved by the Ethics Committee of Universidad Peruana Cayetano Heredia, and all participants signed an informed consent. This study was developed in collaboration with the HIGHCARE-ANDES study.

### Study procedures

All participants underwent thorough clinical examination. Office blood pressure was obtained using a validated oscillometric device (UA-767 Plus; A&D Medical, Japan). Diagnosis of hypertension using office blood pressure used thresholds of SBP ≥140 mmHg or DBP ≥90 mmHg. Average of three measurements performed in seated position after 5 minute rest was considered. Oxygen saturation (SpO_2_) was measured by pulse oximetry (Nellcor N-560, Nellcor Puritan Bennett Inc., USA). Low arterial oxygen saturation was defined as SpO_2_ < 83% (Monge-C et al., 1992). Hct was measured because of its in-field practicality and was determined in duplicate by microcentrifugation using a small blood sample obtained from a puncture on the fingertip. Blood samples were collected after ∼8–10 hour fasting from the antecubital vein, from which serum was obtained for the measurement of glucose, insulin, cholesterol and triglycerides concentrations, and iron profiling. Diabetes and impaired fasting glucose were determined as serum morning glucose >126 mg/dL and >100 mg/dL, respectively. Insulin resistance was determined using the Homeostasis Model Assessment 2 (HOMA2) as HOMA2-IR >1.8. Hypercholesterolemia and hypertriglyceridemia were defined as serum cholesterol >200 mg/dL and triglycerides >150 mg/dL, respectively. In addition, oral glucose tolerance tests were performed in a subset of participants (HH = 27, EE = 27), using a standard method (Aparicio et al., 1974), and the area under the curve was calculated using the trapezoidal rule (Le Floch et al., 1990).

### CVR assessment

The risk of cardiovascular events (stroke, transient cerebral ischemic attacks, myocardial infarction and angina episodes, hospitalization for cardiac decompensation, peripheral artery disease, and cardiovascular mortality) in the following 10 years was calculated for all participants using the Framingham General Cardiovascular Risk Score, which has been validated in Latin America (D'Agostino et al., 2001, 2008). Parameters used for the calculation of the CVR score are age, smoking status, diabetic status, conventional SBP values, total serum cholesterol, and serum high-density lipoprotein (HDL) cholesterol concentrations. High CVR was determined as CVR >20% (D'Agostino et al., 2008).

### Questionnaires

All participants answered a general health and a CMS score questionnaire (León-Velarde et al., 2005). The CMS score measures the severity of the syndrome and is defined based on the following signs and symptoms: EE, shortness of breath or palpitations, sleep disturbances, paresthesias, headache, cyanosis, dilated veins, and tinnitus (León-Velarde et al., 2005). For participants with EE, CMS score was categorized as preclinical (EE only), mild (6–10), or moderate to severe (11–21). Also, a subset of participants completed the international physical activity questionnaire, IPAQ (Craig et al., 2003), from which time in sedentary activities was obtained based on quantity and quality of physical exercise. A trained professional applied all questionnaires.

### Ambulatory blood pressure monitoring

Twenty-four hour ABPM was performed using a validated oscillometric device (TM2430; A&D Medical, Japan) applied on the nondominant arm. The measurements took place every 15 minutes during daytime (6.00–23.00 h) and every 20 minutes during night-time (23.00–6.00 h). Patients were asked to stay still during the recordings and keep a standardized activity journal. Valid ABPM recordings were those with at least 70% of expected readings available and which did not contain two or more consecutive hours without valid readings. Variables obtained from the recordings were systolic, diastolic, and mean daytime (awake), night-time (sleep), and 24 h blood pressure. ABPM thresholds for hypertension were 135/85 mmHg for daytime, 120/70 mmHg for night-time and 130/80 mmHg for 24 h (O'Brien et al., 2013). Also, BP variability, evaluated through standard deviation (SD) of average 24 h, daytime and night-time ABPM values, was analyzed (Mancia et al., 1993).

### Statistical analysis

STATA14 software was used for all statistical analysis. Primary end point was the association between the presence of EE and high CVR; secondary end points were the association between EE and CVR factors individually considered, and differences in ambulatory blood pressure parameters. For comparison of means between groups (HH vs. EE), the normality distribution and homogeneity of variance of all continuous variables were assessed to determine the use of parametric (Student's *t*-test) or nonparametric (Wilcoxon signed-rank) tests. The association between EE and CVR and between EE and cardiometabolic parameters independently was evaluated through multivariate logistic regressions, which included known confounders (age, BMI, and SpO_2_). Physical activity was not included as a potential confounder since there was no association between this parameter and either of the study variables. In addition, bivariate associations were evaluated using logistic regressions. A *p*-value below 0.05 was considered as the minimum level of statistical significance. In addition, a sensitivity analysis was conducted using propensity score matching to determine de effect of EE on CVR. Nearest neighbor with replacement method was used to match subjects with EE and HH whose propensity scores are closer. The method was used to balance the EE and HH groups so that direct comparison would be possible for evaluation of the average treatment effect (ATE) of EE on CVR. The variables included in the calculation of the propensity scores were age, BMI, and SpO_2_.

## Results

### General characteristics

As expected, Hct and CMS scores were higher in participants with EE compared to HH, while SpO_2_ showed lower values. Also, subjects with EE showed higher BMI, heart rate, and age. Regression analysis showed no association between EE and heart rate in a model adjusted for known confounding factors (age, BMI, and SpO_2_); while there was a significant inverse association between heart rate and SpO_2_. Iron profile parameters showed normal values in EE subjects, except for lower serum ferritin. Time spent in sedentary activities or smoking status was not statistically different between HH and participants with EE (Table 1).

**Table 1.** General Characteristics of Healthy Highlanders and Subjects with Excessive Erythrocytosis

	*HH (*n* = 209)*		*EE (*n* = 133)*	
	*Mean*	*SEM*	*Mean*	*SEM*
Age, yrs	40.7	0.9	44.6^**^	1.0
BMI, kg/m^2^	24.9	0.2	26.4^***^	0.3
Time in sedentary activities, hrs	4.0	0.4	5.1	0.5
CMS score	1.8	0.1	7.5^***^	0.3
Hematocrit,%	53.3	0.2	67.4^***^	0.4
Hemoglobin, g/dL	17.8	0.1	22.5^***^	0.1
SpO_2_,%	88.2	0.2	84^***^	0.3
Heart rate, beats/min	67.5	0.7	72.1^**^	1.0
Serum iron, μg/dL	111.3	3.5	105.6	5.5
Serum ferritin, ng/dL	168.1	16.0	125.4^**^	13.7
Serum transferrin, mg/dL	303.1	5.4	308.6	7.1

^**^ *p* < 0.01 versus HH.

^***^ *p* < 0.001 versus HH.

BMI, body mass index; CMS, chronic mountain sickness; EE, excessive erythrocytosis; HH, healthy highlanders; SpO_2_, pulse oxygen saturation.

### Cardiovascular risk

Multivariate logistic regression showed that the odds of CVR >20% in participants with EE 3.6 times the odds in HH, in a model adjusted for overweight and obesity, SpO_2_, and age (Table 2). In addition, a bivariate association was observed between CMS score and CVR. The results from the sensitivity analysis show a statistically significant ATE of EE and CVR (Table 3), supporting the results from the multivariate regression.

**Table 2.** Association Between Excessive Erythrocytosis and Cardiovascular Risk

	*Bivariate analysis*			Adjusted model^*^		
*Variables*	*OR*	*CI 95%*	p *value*	*OR*	*CI 95%*	p *value*
Overweight and obesity			0.132			0.803
No	Reference			Reference		
Yes	1.9	0.8–4.2		1.1	0.41–3.1	
SpO_2_ (%)			0.333			0.729
≥83%	Reference			Reference		
<83%	1.6	0.6–4.2		0.4	0.1–1.5	
Age	1.26	1.16–1.37	0.000	1.28	1.16–1.41	0.000
CMS score			0.0273			
Preclinical	Reference					
Mild	2.7	1.2–6.5				
Moderate and severe	3.5	1.0–11.9				
EE			0.014			0.020
No	Reference			Reference		
Yes	2.7	1.2–6.0		3.6	1.2–10.8	

^*^ Adjusted for overweight and obesity, age, and pulse oxygen saturation (SpO2).

**Table 3.** Average Treatment Effect of Excessive Erythrocytosis on Cardiovascular Risk

*Dependent variable*	*ATE*	*SEM*	p-*Value*	*95% CI*
CVR	0.065	0.027	0.017	0.01–0.11

ATE, average treatment effect; CVR, cardiovascular risk.

### Metabolic parameters

Morning fasting serum glucose concentration was significantly higher in the EE group compared with HH (95.3 vs. 89.8 mg/mL, *p* = 0.019), while serum insulin concentration was similar (9.5 vs. 7.5 μIU/mL). Adjusted multivariate regression showed that the odds of developing insulin resistance in participants with EE were 2.64 times the odds observed in HH (Table 4). In addition, participants with EE showed a greater area under the curve in the oral glucose tolerance test revealing poor glycemic management in these subjects compared with HH (*p* = 0.019). Finally, fasting serum triglycerides were higher in the EE group compared with HH (*p* = 0.012), although no difference between these groups was observed for fasting total serum cholesterol, HDL or LDL.

**Table 4.** Association Between Excessive Erythrocytosis and Systolic and Diastolic Hypertension Measured by ABPM, and Between Excessive Erythrocytosis and Insulin Resistance

	*Bivariate analysis*			Adjusted model^*^		
*Variables*	*OR*	*CI 95%*	p*-value*	*OR*	*CI 95%*	p *value*
Systolic hypertension						
Overweight and obesity			0.001			0.004
No	Reference			Reference		
Yes	6.4	2.1–19.5		5.2	1.7–16.4	
Age			0.023			0.117
<45	Reference			Reference		
≥45	2.8	1.1–7.0		2.1	0.8–56	
SpO_2_ (%)			0.332			0.780
≥83%	Reference			Reference		
<83%	1.7	0.6–4.9		0.8	0.2–2.8	
CMS score			0.010			
Preclinical	Reference					
Mild	3.5	1.3–9.3				
Moderate and severe	4.7	1.3–17.2				
EE			0.001			0.007
No	Reference			Reference		
Yes	5.1	1.9–13.5		4.3	1.5–12.3	
Diastolic hypertension						
Overweight and obesity			0.043			0.143
No	Reference			Reference		
Yes	2.5	1.0–6.1		2.0	0.8–5.0	
Age			0.013			0.043
<45	Reference			Reference		
≥45	3.1	1.3–7.5		2.6	1.0–6.5	
SpO_2_ (%)			0.387			0.832
≥83%	Reference			Reference		
<83%	1.6	0.5–4.6		0.9	0.3–2.8	
CMS score			0.002			
Preclinical	Reference					
Mild	5.1	2.0–12.8				
Moderate and severe	2.0	0.4–10.2				
EE			0.006			0.029
No	Reference			Reference		
Yes	3.5	1.4–8.5		3.0	1.1–8.0	
Insulin resistance						
Overweight and obesity			0.000			0.000
No	Reference			Reference		
Yes	4.9	2.0–11.5		5.6	2.3–13.7	
Age			0.351			0.038
<45	Reference			Reference		
≥45	0.7	0.3–1.4		0.4	0.2–0.9	
SpO_2_ (%)			0.829			0.120
≥83%	Reference			Reference		
<83%	0.9	0.3–2.4		0.4	0.1–1.3	
CMS score			0.076			
Preclinical	Reference					
Mild	2.4	1.1–5.2				
Moderate and severe	1.0	0.2–4.7				
EE			0.011			0.014
No	Reference			Reference		
Yes	2.5	1.2–5.05		2.6	1.2–5.7	

^*^ Adjusted for overweight and obesity, age, and pulse oxygen saturation (SpO_2_).

### Conventional and ambulatory blood pressure

Conventional SBP and DBP values were higher in the group with EE compared with HH. ABPM measurements show that awake SBP, DBP, and mean arterial pressure (MAP) were significantly higher in the EE group. In contrast, during sleep, only DBP was significantly higher in highlanders with EE. Conventional and ABPM values of sea-level (SL) inhabitants are shown for comparison. In agreement with daytime measurements, 24 h SBP, DBP, and MAP were significantly higher in EE participants (Table 5). Moreover, logistic regression analysis showed that the odds of presenting awake systolic and diastolic hypertension measured by ABPM were substantially higher in the EE group compared to HH, adjusted for overweight and obesity, age, and SpO_2_ (Table 4). Additional multivariate regression analysis of a relationship between EE and BP variability as a predictor of CVR could not detect an association between these parameters in our study sample. Finally, we show an additional comparison of the study groups with a sample of SL individuals. This comparison allows for the observation of a gradient of BP values, in which higher values are observed in the SL sample, followed by individuals with EE, and finally the lowest values are found in HH.

**Table 5.** Comparison of Conventional and Ambulatory Blood Pressure Measurements Between Subjects with Excessive Erythrocytosis and Healthy Highlanders from Cerro de Pasco, Peru (4340 m)

	*EE (*n* = 133)*		*HH (*n* = 209)*		*SL (*n* = 40)*	
	*Mean*	*SEM*	*Mean*	*SEM*	*Mean*	*SEM*
Conventional SBP	117.3^*^	1.3	113.5	0.9	116.7	2.0
Conventional DBP	77.4^*^	0.9	75.4	0.7	77.2	1.6
24 h SBP	118.1^*^	1.2	113.7^###^	0.7	123.2	1.4
24 h DBP	74.8^**^	0.8	71.5^###^	0.5	74.5	0.9
24 h MAP	88.6^**^	0.9	85.4^###^	0.5	90.4	0.9
Awake SBP	123.9^***^	1.2	118.8^#^	0.8	127.5^†††^	1.5
Awake DBP	78.9^***^	0.8	75.4^#^	0.5	77.5	0.9
Awake MAP	93.6^***^	0.9	89.5^###^	0.6	93.9	1.0
Sleep SBP	102.1	1.3	100.5^###^	0.9	108.4^†††^	1.4
Sleep DBP	63.8^*^	0.8	61.6^#^	0.6	64.8	1.1
Sleep MAP	76.3	0.9	74.2^###^	0.7	78.6^†^	1.0

Sea-level (SL) values from Lima, Peru (150 m) are shown for comparison.

^*^ *p* < 0.05 versus HH.

^**^ *p* < 0.01 versus HH.

^***^ *p* < 0.001 versus HH.

^#^ *p* < 0.05 versus SL.

^###^ *p* < 0.001 versus SL.

^†^ *p* < 0.05 versus EE.

^†††^ *p* < 0.001 versus EE.

DBP, diastolic blood pressure; MAP, mean arterial pressure; SBP, systolic blood pressure.

## Discussion

### EE and CVR

This is the first study to show an association between the presence of EE and the estimated risk of developing a cardiovascular event in the next 10 years. Our results show that, compared to healthy HA residents, participants with EE have more than three times the odds of being at high (>20%) risk of cardiovascular events over a 10-year follow-up, as estimated by the Framingham General Cardiovascular Risk Score.

Several observational studies at HA have shown an association between EE and metabolic and cardiovascular conditions, including some of those composing the CVR score developed from the Framingham cohort (León-Velarde and Arregui, 1994; Jefferson et al., 2002; Penaloza and Arias-Stella, 2007; Gonzales and Tapia, 2013; Naeije and Vanderpool, 2013; Okumiya et al., 2016). However, to date, no studies on the evaluation of a relatively short-term CVR in HA populations and on its relationship with EE have been published, probably due to the historically few cardiovascular events and low prevalence of CVR factors reported in HA residents worldwide (Hurtado, 1960; Ruiz and Peñaloza, 1977; Faeh et al., 2009; Woolcott et al., 2015). Our findings agree with those from studies at SL, which show a relationship between elevated Hct and the development of cardiovascular diseases (Mcdonough et al., 1965; Goubali et al., 1995), although the mechanisms behind this association are yet to be determined.

### EE and CVR factors: metabolic parameters

At HA, elevated red blood cell count has been associated with metabolic CVR factors such as diabetes, insulin resistance (Okumiya et al., 2011, 2016), and dyslipidemia (Gonzales and Tapia, 2013). Studies in the Andes, China, and India have shown that glucose intolerance is more frequent in patients with elevated Hb compared with those with normal Hb for the altitude of residence. This is in accordance with findings of a relationship between red blood cell count, insulin resistance, and β-cell function observed at SL (Barbieri et al., 2001; Hanley et al., 2009). Conversely, studies on dyslipidemias at HA populations are scarce and inconclusive, and no association between EE and triglyceride levels has been reported so far.

Our findings of an independent association between these CVR factors and the presence of EE are in line with those reported in previous studies, and add further evidence since all risk factors have been measured in the same population. In our study, the presence of EE was associated with higher fasting serum glucose concentration and insulin resistance, and with higher fasting serum triglycerides concentration. In addition, a greater area under the curve in the oral glucose tolerance test in EE participants suggests poor glycemic management in these subjects.

### EE and CVR factors: Blood pressure

While several studies have been published on the effects of EE on pulmonary circulation and the occurrence of cardiovascular events such as right heart failure and pulmonary hypertension (Penaloza and Arias-Stella, 2007; Naeije and Vanderpool, 2013), investigations regarding the systemic circulation in the population with EE are scarce (León-Velarde and Arregui, 1994; Richalet et al., 2005; Maignan et al., 2009). For example, only few studies have reported conventional blood pressure values in highlanders with EE (Richalet et al., 2005; Maignan et al., 2009). In the present study, we report blood pressure parameters measured conventionally and with 24 h ABPM. ABPM is a technique, which allows a more accurate assessment of the actual daily life blood pressure status of an individual than conventional measurements in the doctor's office, and was shown to be particularly useful in assessing blood pressure changes during acute HA exposure (Parati et al., 2014a; Bilo et al., 2015).

Our results of ABPM measurements show a strong association between daytime systolic and diastolic hypertension and the presence of EE. In addition, night-time DBP was found associated with the presence of EE in our study sample. The latter association carries a high predictive value, since at night-time there is no interference from other factors that might affect BP variability such as physical activity, emotional stress and environmental stimulations (O'Brien et al., 2013; Parati et al., 2014b).

Our findings of an association between systemic hypertension and EE using 24 h ABPM agree with the results of previous studies where only conventional BP measurements were used (León-Velarde and Arregui, 1994; Jefferson et al., 2002). Interestingly, at SL, a study by Bertinieri and some of the authors of this article showed in hypertensive patients with primary polycythemia that the reduction of Hct by isovolemic hemodilution resulted in a significant fall in both conventional and 24 h ambulatory blood pressure, supporting the hypothesis of a direct relationship between elevated Hct and hypertension (Bertinieri et al., 1998).

In addition, our results of comparisons with SL values show that HH have lower BP than SL residents, for both daytime and night-time ABPM measurements. These results are in agreement with what has been historically found in observational studies of comparisons in conventional BP values and prevalence of HTA between SL and HA populations (Hurtado, 1960; Marticorena et al., 1969; Negi et al., 2012). The consistency of these observations through different studies suggests that a protection from HTA is present in healthy HA populations, when compared to SL. Interestingly, BP values from the EE group observed in the present study were not significantly different from those found at SL for most ABPM parameters. This supports the hypothesis of a loss of cardiovascular protection in subjects with EE compared with HH.

### EE and CVR factors: Iron profile

Observational studies have suggested that iron profile alterations such as elevated serum iron concentration and ferritin levels might increase the risk of cardiovascular outcomes (Kraml, 2017; Silvestre et al., 2017). Cardiovascular atherosclerosis has been associated with increased iron concentration (Kiechl et al., 1997), and a modest direct relationship has been reported between ferritin levels and metabolic syndrome (Abril-Ulloa et al., 2014). In contrast, a recent meta-analysis of prospective studies shows no association between elevated baseline serum ferritin concentrations and the occurrence of coronary heart disease or myocardial infarction (Das De et al., 2015). These contradictory results indicate that there is still inconsistency in the evidence for the association between iron profile and cardiovascular outcomes. Our results indicate that the higher CVR associated to EE was not related to changes in iron profile, since we found similar iron concentrations in HH and EE subjects, and even lower ferritin values in the EE group; a finding which is in the opposite direction in relationship to the proposed association. Moreover, iron profile mean values were within the reference range, which suggests that lower ferritin levels might be an indicator of a new steady-state of decreased iron stores due to increased erythropoiesis.

### Potential mechanisms of the relationship between EE and CVR

**FIG. 1.**
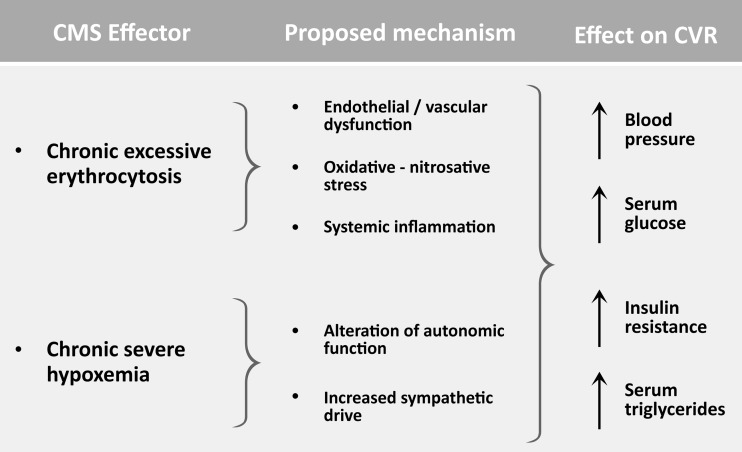
Proposed mechanisms for the association between excessive erythrocytosis and CVR. EE and chronic severe hypoxemia, characteristics of CMS, could contribute to increased CVR through their effects on endothelial and vascular function, oxidative/nitrosative stress, inflammation, and alteration of autonomic function. These effects are reflected in increased BP, altered glucose management, and increased serum lipid levels. CMS, chronic mountain sickness; CVR, cardiovascular risk; EE, excessive erythrocytosis.

Although the mechanisms behind the association between EE and a number of cardiovascular parameters and between the former and CVR are still unclear, some possible explanations for these findings have been suggested. These include an impact of elevated blood viscosity secondary to the EE on several variables involved in cardiovascular function, CVR factors, and CVR, given the major role of red blood cell count as a determinant of blood viscosity (Chien, 1975; Pasquini et al., 1983) as well as an effect of severe hypoxemia on these parameters and on sympathetic activity (Fig. 1).

Rimoldi et al. (2012) evaluated the direct effect of blood viscosity on vascular function at HA by performing hemodilutions in CMS highlanders in the absence of other CVR factors. Before hemodilution, CMS subjects presented vascular dysfunction evidenced by impaired flow-mediated dilation (FMD), arterial stiffness, and greater carotid intima-media thickness compared to HH, suggesting an association between CMS and vascular endothelial dysfunction. Conversely, hemodilution had no detectable effect on vascular function evaluated by FMD. According to the authors, this finding suggests that blood viscosity secondary to EE does not contribute importantly to the observed vascular dysfunction in CMS highlanders. However, their study offers information only on the acute effect of EE on vascular structure and function and not on its chronic effects, which might not be reversible in a short-term follow-up. In a subsequent study, the same research group reported exaggerated oxidative–nitrosative stress in CMS highlanders, which was associated with vascular dysfunction (Bailey et al., 2013). These results support the hypothesis of a detrimental effect of EE on vascular function that might have an impact on CVR. However, further studies are needed to evaluate the chronic effects of EE and the associated elevated blood viscosity on cardiovascular function and on inflammation and damage to other important tissues.

The detrimental effect of the interplay between hemorheological factors, oxidative stress, and inflammation observed on several tissues in animal models suggest that these mechanisms are potential candidates to explain the negative consequences of EE on β-cell function, insulin resistance, and glycemic control in humans. Studies at SL have shown that hyperviscosity and elevated Hct have a negative impact on pancreatic β-cell function and insulin sensitivity, and on glucose disposal rate, affecting blood glucose concentrations (Moan et al., 1994; Irace et al., 2014). Furthermore, high blood viscosity can generate inflammation, fibrosis, and an increment in oxidative stress, causing damage to tissues involved in glucose metabolism (Hanley et al., 2009). Accordingly, a correlation between Hb concentration and insulin resistance has been observed at SL (Barbieri et al., 2001; Hanley et al., 2009), suggesting a role for high blood viscosity on glycemic control.

An additional mechanism contributing to the relationship between EE and CVR is the severely reduced SpO_2_ observed in subjects with EE. This contribution might be through an effect of hypoxemia on CVR factors, independently of EE, or through its impact on sympathetic activity.

Miele et al. (2016) reported a modest independent association between a reduction in SpO_2_, even within normal altitude values range, and the odds of having metabolic syndrome and elevated glycosylated hemoglobin in a cross-sectional sample of adults from Puno, Peru at 3825 m. The results of this study imply that any degree of chronic hypoxemia may worsen metabolic status, which is controversial given findings of cardiometabolic protection conferred by chronic hypoxia to HA dwellers (Baracco et al., 2007; Faeh et al., 2009; Woolcott et al., 2015) in contrast with evidence of the negative effects of severe hypoxemia on cardiometabolic outcomes (Schroeder et al., 2003; Drager et al., 2013; Herrscher et al., 2014).

Rimoldi et al. evaluated the effect of oxygenation on vascular control and showed that oxygen inhalation significantly improved vascular function in hypoxemic highlanders, but it did not normalize these parameters when compared with their values observed in normoxemic HH, suggesting that other mechanisms in addition to hypoxemia might be involved in the observed vascular damage and in explaining its effects on the cardiovascular system (Rimoldi et al., 2012). In our study, we found that the association between EE and CVR, and the association between EE and CVR factors persisted after adjusting for SpO_2_, supporting the hypothesis of additional mechanisms besides hypoxemia contributing to the relationship between these conditions.

Severe hypoxemia in highlanders with EE might also be related to cardiovascular changes through an alteration of autonomic function. A study by Moore et al. suggested an effect of hypoxia on carotid baroreceptor function, with a higher “set-point” for vasoconstrictor stimuli (Moore et al., 2006) in CMS subjects. In addition, Antezana et al. (1995) showed changes in sympathetic activity and increased plasma catecholamines in CMS. Together, these findings suggest a role for altered sympathetic drive associated to differences in CVR between highlanders with EE and healthy HA natives.

Moreover, increased sympathetic activity has been associated with insulin resistance in healthy subjects at SL (Lembo et al., 1994; Reaven et al., 1996). It is possible that this association also contributes to the alterations in glucose metabolism observed in CMS highlanders, and therefore to their cardiometabolic status. However, further studies are needed to define the possible role of autonomic nervous system dysfunction in explaining the relationship between EE and CVR.

### Strengths and limitations

A limitation of the present study is the lack of population-specific threshold values for the diagnosis of risk factors and of CVR in HA dwellers. Those currently available were obtained from analyses of SL populations and developed from international cohorts (WHO, 2007; D'Agostino et al., 2008; Duerden et al., 2015) and thus might not be fully applicable to HA populations, for which further validation is still needed. Nevertheless, since the comparison between groups used the same thresholds, the differences observed maintain validity and are not likely to be affected by the cutoff values. In addition, the CVR score used in this study has been validated for Latin American populations (D'Agostino et al., 2001). However, further prospective studies to validate CVR scores in specific Andean locations at different altitudes are required.

It is also important to mention that the CVR calculated for the present study is a predictor of risk over a relatively short-term follow-up, while it does not estimate the risk of cardiovascular event at any further time point in life. This is important because a recent study in Peru has reported that more than half of the study subjects classified as having low CVR in the short term were at high risk of suffering a cardiovascular event at some point in their lives (Quispe et al., 2015). These findings highlight the relevance of a correct estimation of CVR on different populations, given its impact on the development of public health policies for primary and secondary prevention. This is particularly important in populations with aggravating risk factors, which might be the case of highlanders with EE.

An additional limitation is the inclusion of male participants only. This was due to their higher prevalence of EE compared with women (León-Velarde and Arregui, 1994; Wu et al., 1998), and because of the absence of confounding factors such as menopause, which is well known to have important effects both on erythrocytosis and CVR. In addition, studying male participants allowed for comparison with previous studies in highlanders with EE and CMS. Despite this limitation, the present study offers relevant information to challenge the current knowledge in this field, although its findings need to be confirmed by further epidemiological studies in the Andean HA population.

Finally, the study used a convenience sampling for the recruitment of participants mainly due to its practicality and inexpensiveness, and because in the context of our study aim (association between EE and CVR), the representativeness of the target population is not a requirement as long as the association of interest is maintained. In addition, given that both study groups have been selected through the same method, no differential selection between study groups was expected. Regarding internal validity, convenience sampling could lead to unbalances between the exposed and unexposed groups in terms of potential confounders that could generate biased results. To control for potential confounding in our study, we have used multiple regression and a sensitivity analysis using propensity score matching to test for the robustness of the association.

## Conclusions

In conclusion, our results show that the odds of developing cardiovascular events in the next 10 years is higher in subjects with EE compared with HH, and that the odds is even higher in the subgroup of highlanders with moderate to severe CMS, compared with mild CMS.

In addition, we found an association between CVR factors, independently considered, and the presence of EE. In separate regression models, we show higher odds of increased CVR associated with an elevation of daytime systolic and diastolic hypertension, of fasting glycemia and insulin resistance, and of fasting serum triglyceride concentration in highlanders with EE compared with HH.

The observed associations might be explained by the detrimental effects of elevated blood viscosity and EE on several variables involved in cardiovascular and metabolic function, which might indirectly increase CVR, in addition to its direct impact on the development of cardiovascular events. However, further studies are needed to evaluate the mechanisms of the chronic effects of EE and the associated elevated blood viscosity on CVR, and of the direct or indirect effects of severe hypoxemia on cardiovascular function.

Overall, our results indicate that highlanders with EE are at higher risk of suffering from cardiovascular disease, suggesting that the HA-induced protection against cardiovascular and metabolic diseases is not present in Andeans with EE. Therefore, our findings highlight the necessity of regular control of glycemia and insulinemia (or insulin sensitivity tests), blood lipids, and blood pressure evaluation by ABPM, in addition to the usual regular (6–12 months) Hct and CMS score check-ups, to provide adequate healthcare to highlanders with EE.
